# Shiga Toxins: An Update on Host Factors and Biomedical Applications

**DOI:** 10.3390/toxins13030222

**Published:** 2021-03-18

**Authors:** Yang Liu, Songhai Tian, Hatim Thaker, Min Dong

**Affiliations:** 1 Department of Nephrology, The First Hospital of Jilin University, Changchun 130021, China; 2 Department of Urology, Boston Children’s Hospital, Boston, MA 02115, USA; Songhai.Tian@childrens.harvard.edu (S.T.); hatim.thaker@childrens.harvard.edu (H.T.); 3 Department of Microbiology and Department of Surgery, Harvard Medical School, Boston, MA 02115, USA

**Keywords:** Shiga toxin, EHEC, *Shigella*, hemolytic uremic syndrome, Gb3, LAPTM4A, TM9SF2, immunotoxin, bacterial toxins, toxins

## Abstract

Shiga toxins (Stxs) are classic bacterial toxins and major virulence factors of toxigenic *Shigella dysenteriae* and enterohemorrhagic *Escherichia coli* (EHEC). These toxins recognize a glycosphingolipid globotriaosylceramide (Gb3/CD77) as their receptor and inhibit protein synthesis in cells by cleaving 28S ribosomal RNA. They are the major cause of life-threatening complications such as hemolytic uremic syndrome (HUS), associated with severe cases of EHEC infection, which is the leading cause of acute kidney injury in children. The threat of Stxs is exacerbated by the lack of toxin inhibitors and effective treatment for HUS. Here, we briefly summarize the Stx structure, subtypes, in vitro and in vivo models, Gb3 expression and HUS, and then introduce recent studies using CRISPR-Cas9-mediated genome-wide screens to identify the host cell factors required for Stx action. We also summarize the latest progress in utilizing and engineering Stx components for biomedical applications.

## 1. Introduction

Shiga toxin (Stx) was named after Japanese microbiologist Kiyoshi Shiga, who identified and characterized *Shigella dysenteriae* in 1897 [1]. Among *Shigella dysenteriae* strains, serotype 1 is the toxigenic one that expresses Stx [2]. In 1977, a cytotoxic toxin from *Escherichia coli* (*E. coli*) isolates was discovered by Konowalchuck et al., initially named verotoxin or *E. coli* cytotoxin for its ability to kill cultured Vero cells [3]. By the early 1980s, O’Brien et al. recognized that *E. coli* isolates express toxins highly similar to Stx; they called them Shiga-like toxins and designated the *E. coli* isolates Shiga-like toxin-producing *E. coli* (STEC) [4]. Among STEC, the strains associated with human diseases are also known as enterohemorrhagic *E. coli* (EHEC). It was soon realized that these toxins belong to the same Stx family and can be divided into two serotypes: Stx1 is almost identical to the prototype Stx in *Shigella dysenteriae*, while Stx2 shares ~56% protein sequence identity with Stx [5].

Each year, in the United States, there are approximately 265,000 cases of EHEC infection [6], which usually starts with diarrhea and can develop into dysentery and hemorrhagic colitis. In severe cases, life-threatening complications such as hemolytic uremic syndrome (HUS) and neurological disorders can occur. HUS is most commonly associated with EHEC serotype O157:H7 infections, and Stx is the major cause [7]. HUS has the highest incidence in children and the elderly [8,9] and is the major reason for acute kidney injury in children [10]. Patients have typically developed irreversible vascular damage by the time symptoms appear, and there is no specific treatment [7].

Investigations over the past 40 years have elucidated the molecular mechanism underlying the toxicity of Stx, established its central role in pathogenesis, and shed light on many key cellular functions. Furthermore, Stx and Stx fragments have also been utilized as tools for biomedical applications. Many topics in Stx biology and applications have been covered by excellent in-depth reviews in recent years [9,11,12,13,14,15,16,17,18,19,20,21,22]. Here, we will briefly introduce the mode of action for Stx and then focus on the latest progress in identifying host factors and engineering Stx for biomedical applications.

## 2. Stx Structure and Function

**Figure 1:**
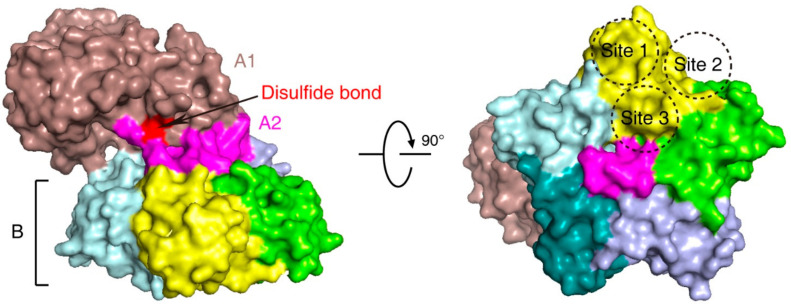
The structure of Shiga toxin: an A subunit, which is cleaved into an enzymatic piece A1 (colored brown) and a linker piece A2 (colored magenta), and five B subunits (PDB: 4M1U). The disulfide bond connecting A1 and A2 pieces is colored red. In the bottom view (right panel), three receptor binding sites on one B subunit (colored yellow) are shown.

Stx is an AB5 toxin, comprising an enzymatic A subunit (Stx-A, 32 kDa) and five identical B subunits (Stx-B, 7.7 kDa) that form a pentamer. The A subunit connects to the pentamer noncovalently by inserting its C-terminal region into the central hole [23,24] (Figure 1). The A subunit is an RNA *N*-glycosidase, which inhibits protein synthesis by cleaving 28S ribosomal RNA [25,26,27]. The B subunit is responsible for binding to receptors. The glycosphingolipid globotriaosylceramide (Gb3, also known as CD77) is the major receptor of Stx [28]. Crystal structural studies using Stx1-B and a trisaccharide analog of Gb3 revealed three Gb3 binding sites per B subunit (Figure 1). Thus, one Stx can bind up to 15 Gb3 simultaneously [29]. After clustering Gb3 on cell surfaces, Stx is internalized by clathrin-dependent and -independent endocytosis pathways [30,31], retrogradely sorted into the *trans-*Golgi network (TGN) and further into the endoplasmic reticulum (ER), bypassing the late endocytic pathway [32,33,34,35,36]. The A subunit is further processed by host furin and furin-like proteases (cleaving between R251 and M252 of Stx1 and between R250 and A251 of Stx2) into the enzymatic piece A1 (27.5 kDa) and the linker piece A2 (4.5 kDa), connecting to the B subunit. The A1 and A2 domains remain connected by a single disulfide bond, which is reduced within the ER. The A1 domain is then released from the ER into the cytosol through the ER-associated protein degradation (ERAD) pathway to inhibit protein synthesis [14,37,38].

## 3. Stx Subtypes

Stx1 and Stx2 each have multiple subtypes classified based on protein sequence variations. Three subtypes of Stx1 (Stx1a, Stx1c, and Stx1d) and eleven subtypes of Stx2 (Stx2a–Stx2k) have been reported so far [39,40,41,42]. Some STEC strains express only one type of Stx, while others may express multiple types simultaneously [39]. Protein stability, toxin potency, receptor preference, symptom severity, and related host reservoirs may vary among subtypes [39,43]. For example, strains that produce Stx2a, Stx2c, and Stx2d are often associated with colitis and HUS in human infections. STEC producing Stx2b, Stx2e, Stx2f, and Stx2g are usually associated with animal infections, such as in deer, pigs, pigeons, and cattle [17,41,44,45]. Stx2e is the common subtype causing the edema disease of swine [46]. Strains producing Stx2f are frequently found in pigeons and other birds [47,48,49]. In 2018, Stx2h and Stx2i were identified in STEC strains isolated from wild marmots and shrimp, respectively [40,50]. Two additional new subtypes, Stx2j and Stx2k, have recently been reported but have yet to be broadly accepted. Stx2k was isolated from bacterial strains of various sources, including animals (goats and pigs), raw meat (beef and mutton), and human patients with diarrhea [41,42].

Stx1 and most Stx2 subtypes recognize the Gb3 receptor. However, Stx2e binds preferentially to globotetraosylceramide (Gb4), which is synthesized by adding N-acetylgalactosamine to Gb3 [51]. Toxicity differences among Stx subtypes are partially ascribed to differences in receptor binding, and it is believed that the low dissociation rate of Stx2a with receptors may lead to greater toxicity due to longer toxin uptake [52]. This may be one reason why Stx2a, among all Stx2 subtypes, is implicated in most HUS cases [53].

## 4. In Vitro Cultured Human Cell Models and In Vivo Animal Models

A variety of human cell lines and primary cells have been utilized in studying Stx, such as colorectal adenocarcinoma cell lines HT-29 [54], Caco-2 and HCT-8 [55], glomerular endothelial cells [56], renal tubular epithelial cells [57], vascular endothelial cells [58], cervical cancer HeLa cells [59], Burkitt lymphoma cells [60], and macrophage-like THP-1 Cells [61]. Some cell lines can be sensitized to Stx by ectopic expression of exogenous Gb3 synthase [62,63].

Tian et al. recently reported that the bladder cancer cell line 5637 was the most sensitive to Stx among a group of human cancer cell lines, with toxin doses that induce 50% of cell death at 0.028 (Stx1) and 0.007 (Stx2) ng/mL, respectively [64]. This is because 5637 cells endogenously express high levels of Gb3 [64]. This cell line could serve as a convenient and sensitive human cell line model for investigating Stx action and developing Stx inhibitors.

The sensitivity of different cell lines to Stx depends not only on Gb3 expression levels but also on membrane microdomains, as well as other host factors involved in toxin trafficking [14,62,65,66,67,68]. For example, ACHN cells and Caki-2 cells, which are two human renal tubular adenocarcinoma cell lines, showed drastically different levels of sensitivity to Stx2: ACHN cells are highly sensitive while Caki-2 cells are not. Further analysis shows that they have similar Gb3 levels. RNA sequencing analysis of the genes differentially expressed by ACHN and Caki-2 cells identified RAB5A, TRAPPC6B, and YKT6 as host cell factors required for the high sensitivity of ACHN to Stx2 [68].

As EHEC infection occurs in the intestine, human intestinal organoids (HIOs) have recently been utilized as a physiologically relevant model to investigate the effects of Stx on human intestinal cells. HIOs can be derived from pluripotent stem cells or intestinal stem cells and represent the small intestinal tissues to which EHEC attaches [69,70]. Pradhan et al. showed that HIOs express Gb3 as well as the transcripts of Gb3 synthase. Injection of Stx2a into HIOs in vitro induced necrosis and the apoptotic death of cells. HIOs can also be transplanted into mice, providing a method to assess the impact of Stx on human intestinal tissues in vivo [71].

Various animal models have been established for studying EHEC infection and Stx [72], including mice, rats, infant rabbits, and nonhuman primates [12,16]. In mice, Gb3 expression has been reported in the cerebral cortex, the microvascular endothelial cells of the pia mater, and renal tubular capillaries. However, there is no expression in the renal vascular endothelium, and mice do not develop HUS with Stx injections [73,74]. A HUS model of C57BL/6 mice can be established using a combination of Stx2 and lipopolysaccharide (LPS) via intraperitoneal (i.p.) injection [75]. These mice develop thrombocytopenia, hemolytic anemia, as well as renal failure, similar to HUS presentation in humans [75]. Previous studies have suggested that the NLRP3 (NOD-, LRR-, and pyrin domain-containing protein 3) inflammasome pathway can be triggered by Stx, leading to proinflammatory IL-1β release and cell death [76]. However, this inflammasome activation was later found not to be mediated by Stx itself but by LPS contamination copurified with Stx [77]. Though EHEC cannot normally infect mice [78,79,80,81], an alternative model has been developed by expressing Stx in *Citrobacter rodentium*, a native mouse pathogen [82]. A recent study showed that LPS-mediated inflammasome responses are inhibited by Stx in Stx-expressing *C. rodentium* infection in a mouse model, suggesting that Stx contributes to suppression of host defense against bacteria [77].

In addition to mice, rats have also been utilized as a model. Stx i.p. injection causes acute tubular necrosis, polyuria, and reduced urinary osmolality in rats as well as an increase of aquaporin type 2 and β2-microglobulin in urine, indicating the dysfunction of water reabsorption in proximal and collecting tubules [83]. Adult rats i.p. injected with culture supernatant from recombinant *E. coli* expressing Stx2 (sStx2) can develop watery diarrhea, thrombocytopenia, hemolytic anemia, glomerular thrombotic microangiopathy, and tubular injury, which may result from the combined effect of Stx2 and other bacterial factors in the culture supernatant [84]. However, due to the low expression of Gb3 in vascular endothelial cells in rodents, weaned rats treated with sStx2 show few glomerular thrombotic microvascular lesions associated with HUS, despite the observation of glomerular mesangial hyperplasia [85].

Gb3 is expressed in the colon of infant rabbits [86]. After oral administration of Stx2, three-day-old New Zealand white rabbits showed diarrhea and intestinal inflammation but no HUS symptoms [16]. Either intravenous injection of Stx2 (1200 ng/kg) or oral administration of EHEC can cause diarrhea as well as intestinal and renal histological damage in weaned Dutch Belted (DB) rabbits [87,88].

The distribution and expression of Gb3 in primates such as macaques and baboons may resemble that of humans [89,90,91,92]. A baboon model has been created by intravenous administration of Stx (50–200 ng/kg), which elicited thrombocytopenia, microangiopathic hemolytic anemia, and glomerular damage, similar to what is observed in Stx-mediated HUS in humans [91,93].

## 5. Gb3 Expression in Humans and Hemolytic Uremic Syndrome (HUS)

In humans, Gb3 expression has been reported in renal epithelium and endothelium, microvascular endothelial cells in the lamina propria of the intestine, intestinal pericryptal myofibroblasts, and subpopulations of B-lymphocytes in germinal centers [13,14]. Gb3 has also been found in smooth muscle cells of the digestive tract, the urogenital system, the placenta [94,95], endothelial cells, dorsal root ganglion cells in the peripheral nervous system [14,96], and endothelial cells and neurons in the central nervous system [14,97]. Gb3/CD77 is also known by two other names: (1) Burkitt lymphoma antigen, as it is found in many Burkitt lymphoma cancer cells [98], and (2) P^k^ blood group antigen, which belongs to the human P1PK blood group system consisting of three glycosphingolipid antigens (P^K^, P1, and NOR). P^K^ antigen is expressed in red blood cells in most individuals [14,94]. It has been suggested that Stx can bind to blood cells and cause complement-mediated hemolysis [99]. Interestingly, microvesicles shed by blood cells may contain Stx, which has been suggested to mediate the circulation and absorption of toxins into endothelial and epithelial cells in the kidney [100].

HUS is a severe thrombotic microangiopathy characterized by renal injury, microangiopathic hemolytic anemia, and thrombocytopenia [101]. HUS caused by EHEC infection is known as diarrhea-positive HUS (D+HUS) or typical HUS, while atypical HUS can be caused by other infections, complement disorders, or other genetic mutations [102]. EHEC-HUS initially presents with symptoms of hemorrhagic colitis and subsequently includes hemolytic anemia, thrombosis, and kidney injury, leading to weakness, shortness of breath, skin ecchymosis, oliguria, edema, hypertension, and serious neurological symptoms such as epilepsy [9].

Microvascular endothelial cell apoptosis induces edema, while platelet and fibrin accumulation can further impede blood flow [103,104]. Platelets are reduced, partly due to microthrombi deposition along the endothelial cell wall and consumption by the reticuloendothelial system [105]. Thrombocytopenia may also result from Stx binding to activated platelets and immature megakaryoblasts in the bone marrow, inducing their apoptosis [106,107]. Mechanical destruction of erythrocytes through damaged blood vessels leads to hemolytic anemia, which combines with reduced microcirculation to produce multisystem ischemia [104]. It has also been proposed that Stx-triggered host immune responses in the gut and activation of immune cells may be another key contributor to the development of HUS [108]. Current EHEC-HUS treatment relies on supportive therapy. This includes fluid resuscitation, correcting electrolyte abnormalities, and controlling hypertension [34]; blood transfusions and renal replacement therapy are often required [105,109].

## 6. Host Factors Recently Identified through CRISPR-Cas9 Screens

The CRISPR (clustered regularly interspaced short palindromic repeats)-Cas9 system can be viewed as a form of acquired immune system in bacteria against invading genetic elements. Cas9 is an endonuclease that uses guide RNA sequences encoded in CRISPR to recognize and cleave specific target DNA [110]. This system has now become a powerful technological platform to easily modify genes in eukaryotic cells. In 2014, Shalem et al. and Wang et al. developed genome-scale CRISPR-Cas9-mediated knockout screening in human cells [111,112]. Using this powerful approach, four groups independently carried out genome-wide CRISPR-Cas9 mediated screens to identify Stx host factors [64,113,114,115]. Although different cell lines and guide RNA libraries were utilized, the host factors identified from these screens are largely involved in the biosynthesis of Gb3, indicating that recognition of Gb3 is the major rate-limiting step in Stx intoxication.

**Figure 2:**
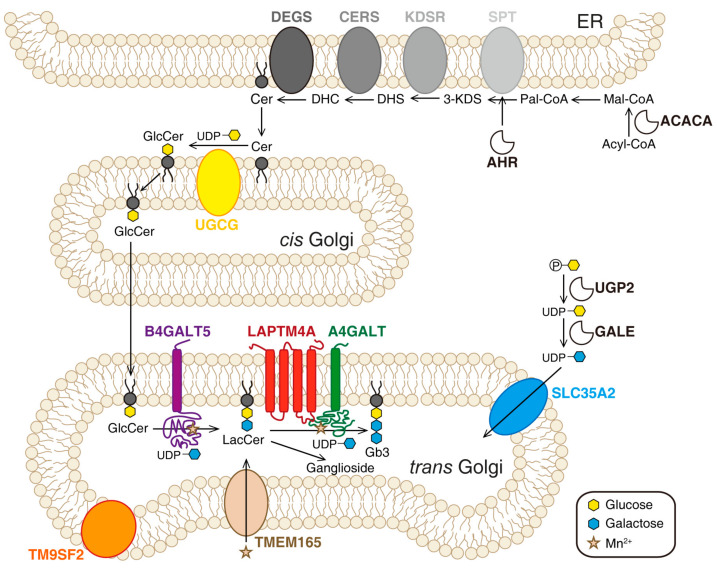
CRISPR (clustered regularly interspaced short palindromic repeats)/Cas9 screens identified genes involved in Gb3 biosynthesis, with a schematic diagram of the Gb3 biosynthesis pathway and a summary of the top genes identified in CRISPR/Cas9 screens. Cer: ceramide. ACACA: acetyl-CoA carboxylase alpha. Acyl-CoA: acetyl-CoA. Mal-CoA: malonyl-CoA. Pal-CoA: palmitoyl-CoA. SPT: serine palmitoyl transferase complex. 3-KDS: 3-dihydrosphingosine. DHS: dihydrosphingosine. KDSR: 3-ketodihydrosphingominol reductase. DHC: dihydroceramide. CERS: ceramide synthase family enzymes. DEGS: dihydroceramide Δ4-desaturases. GlcCer: glucosylceramide. UGCG: ceramide glucosyltransferase. LacCer: lactosylceramide. B4GALT5: *β*-1,4-galactosyltransferase 5. A4GALT: *α*-1,4-galactosyltransferase. UGP2: UDP-glucose pyrophosphorylase 2. GALE: UDP-galactose 4-epimerase. SLC35A2 transports UDP-galactose from the cytosol into Golgi. Four new host factors are shown: LAPTM4A (lysosomal-associated protein transmembrane 4 alpha) may be involved in the synthesis of Gb3 from LacCer; TM9SF2 (transmembrane 9 superfamily member 2) and TMEM165 (transmembrane protein 165) may play a role in maintaining a suitable environment in the Golgi for optimal glycosyltransferase activity; AHR (aryl hydrocarbon receptor) can directly bind and activate the promoter of the gene encoding serine palmityl transferase subunit A (SPTSSA), which regulates the first step in the biosynthesis of new sphingolipids.

Multiple ER- and Golgi-localized enzymes are involved in the biosynthesis of Gb3 (Figure 2). Ceramide (Cer) is the precursor of glycosphingolipids. The de novo synthesis of Cer happens on the cytosolic side of the ER membrane. Serine palmitoyl transferase (SPT) complex catalyzes the conversion of serine and palmitoyl-CoA (Pal-CoA) into 3-dihydrosphingosine (3-KDS). Then, 3-KDS is converted to dihydrosphingosine (DHS) under the catalysis of 3-ketodihydrosphingominol reductase (KDSR). A range of acyl-CoA with saturated or unsaturated fatty acid chains can be subsequently incorporated into DHS to produce dihydroceramide (DHC). This step is catalyzed by ceramide synthase family enzymes (CERS). DHC is further oxidized to Cer by dihydroceramide Δ4-desaturases (DEGS). The glycosylation of Cer occurs on Golgi. First, one glucose residue is transferred onto Cer to produce glucosylceramide (GlcCer). This reaction is catalyzed by ceramide glucosyltransferase (UGCG) on the cytosolic side of *cis*-Golgi. Then, GlcCer is flipped into the lumen side and transported to the *trans*-Golgi. Two galactose residues are transferred to GlcCer to produce lactosylceramide (LacCer) and Gb3 sequentially. These steps are catalyzed by *β*-1,4-galactosyltransferase 5 (B4GALT5) and the Gb3 synthase known as *α*-1,4-galactosyltransferase (A4GALT), respectively [116,117,118]. SLC35A2 (UDP-galactose translocator) transports active galactose into the lumen of Golgi [64].

In 2018, Tian et al. reported genome-wide CRISPR-based loss-of-function screens for Stx1 and Stx2 using 5637 cells [64]. The same year, Pacheco et al. performed a screen for EHEC infection using HT29 cells [113]. In 2019 and 2020, two more screens for Stx using HeLa cells were published [114,115]. All four screens identified major players in the Gb3 biosynthesis pathway, such as A4GALT, SPTSSA, UGCG, B4GALT5, and SLC35A2. Three novel factors were then identified: LAPTM4A (lysosomal-associated protein transmembrane 4 alpha), TMEM165 (transmembrane protein 165), and TM9SF2 (transmembrane 9 superfamily member 2). In addition, the screen by Majumder et al. also identified aryl hydrocarbon receptor (AHR), a ligand-activated transcription factor, which might be required for the expression of several genes involved in the Gb3 biosynthesis pathway [115]. Tian et al. further carried out parallel screens using the plant toxin ricin, whose trafficking pathway and mode of action are similar to those of Stx but utilizes a broad range of glycans as receptors. The results indicate that LAPTM4A is specific to Stx, whereas TMEM165 and TM9SF2 are also required for ricin [64].

LAPTM4A is ranked as a top hit in all four screens and has never been previously implicated as a host factor for Stx [64,113,114,115]. LAPTM4A knockout (KO) renders cells completely resistant to Stx1 and Stx2, but not other control toxins (ricin and cholera toxin). The resistance of LAPTM4A KO cells is at the same level as A4GALT KO cells. LAPTM4A is a poorly characterized small (233 amino acids) 4-transmembrane protein with unknown functions [119,120]. Tian et al. carried out mass-spectrometry-based lipidomic analysis and found that Gb3 is greatly reduced in LAPTM4A KO cells and the levels of Gb3 precursor LacCer are increased [64]. Yamaji et al. obtained similar results using thin-layer chromatography and radiography analysis [114]. These results demonstrate that LAPTM4A is essential for the last step of Gb3 biosynthesis.

LAPTM4A was initially reported to be localized on endosomes and lysosomes. However, the localization of LAPTM4A appears to be sensitive to a tag added to the N-terminus in these earlier studies [120,121,122]. Tian et al. found that LAPTM4A with a C-terminal tag was largely localized to Golgi in three different human cell lines [64]. The endogenous LAPTM4A has been detected in both the Golgi and lysosome fractions of rat liver membrane lysates [119,120]. These findings indicate that Golgi is at least one of the places containing LAPTM4A. Tian et al. also established the membrane topology of LAPTM4A, demonstrating that its N- and C-termini are both located in the cytosol, with two short luminal domains. Coimmunoprecipitation assays have shown that LAPTM4A can interact with A4GALT, but the expression level and Golgi-localization of A4GALT are not affected in LAPTM4A KO cells [64].

Both Tian et al. and Yamaji et al. showed that the requirement of LAPTM4A for Gb3 synthesis is not shared by its homolog LAPTM4B [64,114]. By constructing and evaluating a series of chimeric proteins between LAPTM4A and LAPTM4B, Tian et al. determined that the second luminal domain of LAPTM4A is required for its role in Gb3 synthesis. Based on these findings, Tian et al. proposed that LAPTM4A serves as a specific “activator” for A4GALT [64]. Supporting that suggestion, Yamaji et al. found that Gb3 synthesis activity in cell lysates of LAPTM4A KO cells is greatly reduced compared with control cell lysates. They also found that exogenously expressed A4GALT levels are not affected in LAPTM4A KO cells, although endogenous A4GALT levels in HeLa cells have been difficult to detect [114]. These findings clearly suggest that LAPTM4A is an essential cofactor for A4GALT [64,114]. Future studies are required to further elucidate whether LAPTM4A is required for A4GALT enzymatic activity and/or its stability. The molecular mechanism underlying LAPTM4A-A4GALT interactions also remains to be established.

In contrast with the specific requirement of LAPTM4A for Gb3 biosynthesis, TM9SF2 and TMEM165 are required for a broad range of glycosylation processes, including biosynthesis of gangliosides and heparan sulfate proteoglycan. They have been identified in a growing number of genetic screens for various toxins and viruses [123,124,125,126,127]. Mutations in TEME165 have been linked to congenital disorders of glycosylation in humans [128]. TMEM165 is a multitransmembrane protein, localized on Golgi, that has been proposed to act as an Mn^2+^ transporter to maintain Mn^2+^ homeostasis [129]. Since Mn^2+^ is an essential cofactor for many glycosyltransferases in Golgi, TMEM165 is required for the biosynthesis of both glycoproteins and glycolipids. Consistently, Tian et al. found that TMEM165 KO cells have low levels of not only Gb3 and Gb3 precursors but also gangliosides. The addition of Mn^2+^ to culture medium restored the binding of Stx to TMEM165 KO cells, which is consistent with the role of TMEM165 in regulating Mn^2+^ hemostasis [64,128,129,130].

The function of TM9SF2 remains unknown. It contains nine transmembrane domains and belongs to a family of highly conserved membrane proteins, with four members in humans (TM9SF1 to TM9SF4). Yamaji et al. showed that Stx binding of TM9SF2-KO cells can be recovered by all TM9SF family members, suggesting that TM9SF2 family members regulate Gb3 synthesis by a conserved mechanism [114]. TM9SF2 was recently identified as a potential oncogene in colorectal cancer through transposon mutagenesis in mouse models and found to be overexpressed in many human colorectal cancer samples [131]. Although it was initially suggested to be an endosomal protein, recent studies using antibodies capable of recognizing endogenous TM9SF2 revealed that it is largely localized to the Golgi [113,124]. TM9SF2 KO cells showed a reduction in glycosylation of all glycolipids and glycoproteins, including heparan sulfate proteoglycan [124]. TM9SF2 KO cell lysates showed levels of A4GALT activity comparable with control lysates, further suggesting that TM9SF2 is not directly involved in A4GALT enzymatic activity in vitro [113,124,132]. The exact mechanism by which TM9SF2 affects glycosylation remains unknown and could be multifaceted. It is possible that TM9SF2 acts as a transporter for maintaining the hemostasis of some, as yet unidentified, elements critical for glycosylation, similar to TMEM165. It is also possible that TM9SF2 is involved in Gb3 biosynthesis by regulating retrograde trafficking of lipids and glycosyltransferases [64,114]. Tian et al. found that TM9SF2 KO cells appear to form large vacuoles in the cytosol and showed severely disrupted endosomal trafficking of exogenously loaded lipids [64]. This effect on endosomal trafficking has been shown for TM9SF2 paralogs in both *Drosophila* and yeast, suggesting that TM9SF2 plays a conserved role in this process [133,134]. As TM9SF2 is localized on the Golgi, the defects in endosomal trafficking could be secondary, although it remains possible that a low level of TM9SF2 is also localized on endosomes.

The screen by Pacheco et al. is unique in that it was carried out using EHEC instead of purified Stx [113]. Besides Stx, EHEC also exhibits its virulence via a type III secretion system (T3SS), which injects a group of effectors into host cells to disrupt cell functions. The cell line used in the screen, HT-29, is not sensitive to purified Stx, but incubation with EHEC resulted in cell death via effectors injected through the T3SS. The screen was designed and carried out with the intention of identifying host factors required for T3SS virulence. Surprisingly, the top hits were all involved in the Gb3 biosynthesis pathway, suggesting that Gb3 is critical for the virulence of the T3SS, in addition to its role as the receptor for Stx, although the mechanism remains to be determined [113]. These findings further illustrated the importance of the Gb3 biosynthesis pathway as a therapeutic target for developing inhibitors against Stx and EHEC [64,113,114,115].

## 7. Biomedical Application of Shiga Toxin Subunit A

Protein toxins such as Stx are evolved to achieve both specific targeting of host cells and efficient delivery of a payload into the cytosol of target cells. These properties could be utilized for developing novel therapeutic proteins. Rapid progress has been made on utilizing Stx-A and Stx-B in recent years. Stx-A has been selected as a promising payload in constructing immunotoxins for targeting and killing cancer cells. Immunotoxins are fusion proteins composed of a toxic domain such as Stx-A and a targeting domain, which can be single-chain antibody fragments, nanobodies, or any other protein binders that can specifically recognize cell surface proteins expressed in cancer cells [135]. The concept of immunotoxins was proposed and explored in the 1970s [136], but this approach has encountered significant difficulties, including lack of specificity in targeting cancer cells, high systemic toxicity, and generation of neutralizing antibodies that render the immunotoxin ineffective. Fortunately, many of these challenges can now be addressed. For instance, highly specific cell surface proteins on myeloma cells have been validated, and specific antibodies against them developed [137]. Stx-A is an ideal payload for this purpose: (1) it is highly potent and targets 28S rRNA—as a foreign protein, it would be difficult for cancer cells to develop resistance to Stx-A; (2) the population is not immunized against Stx; (3) it is relatively a small protein (32 kDa), which facilitates protein engineering.

**Figure 3:**
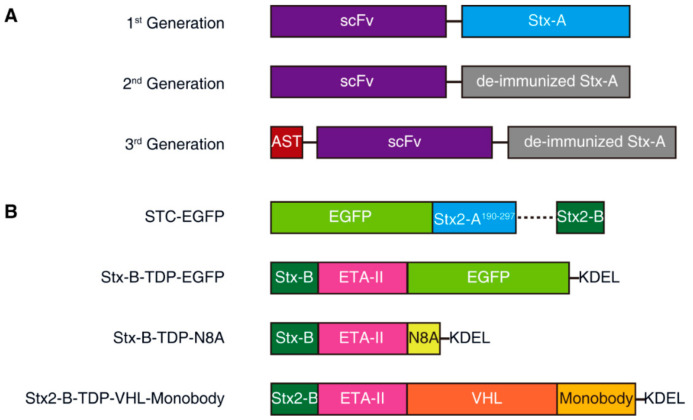
Schematic diagrams of engineering Stx-A and Stx-B for biomedical applications. (**A**) Three representative generations of engineered toxin bodies (ETBs) constructed with wild-type Stx-A (1st generation) or deimmunized Stx-A (2nd and 3rd generations). scFv, single chain variable fragment; AST, antigen seeding technology (e.g., viral peptide is derived from cytomegalovirus). (**B**) STC (Shiga-like toxin-based carrier) is a delivery system in which the Stx2 A1 piece is replaced with delivery cargoes like EGFP. Stx-B-TDP is a delivery system fusing Stx-B with the *P. aeruginosa* exotoxin A translocation domain (ETA-II) and cargoes, e.g., EGFP or N8A (an MDM2 inhibitor), at its C-terminus. Another design, e.g., the Stx-B-TDP-VHL-monobody, fuses ETA-II and cargoes (Von Hippel-Lindau (VHL) fusion with a monobody) at the C-terminus of Stx-B. An additional ER retention sequence (KDEL) is added for retrograde transport.

Exciting progress in utilizing Stx-A has been made by a biotech company, Molecular Template, which has been developing Stx-A-based immunotoxins (called engineered toxin bodies (ETBs)). Three generations of immunotoxins have been developed and tested. The first generation (MT-3724) was based on wild-type Stx2-A fusion with a single-chain variable fragment (scFv) derived from an antibody targeting CD20, which is a B-cell surface antigen, targeted to treat B-cell lymphomas and leukemias (Figure 3A). This ETB is currently undergoing multiple Phase 2 studies to show monotherapy activity. The major side-effect is capillary leak syndrome, which is likely due to immune responses against ETB proteins [138,139,140].

The second-generation ETB utilized a modified Stx-A containing mutations to reduce immunogenicity (deimmunization, Figure 3A). A representative mutation, TAK-169, was created by the fusion of deimmunized Stx-A with an scFv targeting CD38, which is a marker for myeloma cells. Encouraging data on nonhuman primates have shown that TAK-169 can be dosed at higher levels than MT-3724 and can induce lower levels of immune response compared with MT-3724 [141,142]. In 2019, through a collaboration with Takeda Pharmaceutical, a Phase 1 first-in-human, multicenter, open-label study of TAK-169 began enrolling patients in an evaluation of safety, drug resistance, initial efficacy, pharmacokinetics (PK), and pharmacodynamics (PD) [142].

Another second-generation lead protein, MT-5111, incorporates a different scFv, targeting HER2. The binding site of MT-5111 to the HER2 domain is designed to be different from that of trastuzumab and pertuzumab, two commonly used antibodies against HER2-positive tumors. In the presence of these monoclonal antibodies, MT-5111 can still bind to HER2 and induce effective cell killing. The Phase 1 clinical trial of MT-5111 is enrolling patients with HER2-positive solid tumors that have progressed after receiving other approved treatments [143,144].

The third-generation ETB further includes the fusion of both a viral foreign peptide and deimmunized Stx-A to an scFv targeting PD-L1 (Figure 3A). The viral peptide is derived from cytomegalovirus (CMV). Once delivered into target tumor cells, the viral peptide will be presented as a viral antigen and can activate CMV-reactive T-cells to recognize and destroy tumor cells. This technology is known as “antigen seeding”; this third-generation ETB is undergoing preclinical testing [145,146].

## 8. Biomedical Application of Shiga Toxin Subunit B

Stx-B is an ideal tool for targeting Gb3-expressing cells. In healthy humans, Gb3 expression is highly restricted to only a few cell types, including dendritic cells [13]. As dendritic cells are primary antigen-presenting cells and play a critical role in inducing T-cell responses against viral pathogens and tumors, Stx-B has been explored to deliver antigens into dendritic cells for antigen presentation [147,148]. Haicheur and Benchetrit et al. discovered that Stx-B conjugated with ovalbumin (OVA), a full-size antigenic model protein, efficiently delivers exogenous peptides into the MHC class I- and class II-restricted delivery pathways of mouse dendritic cells. This pathway induces humoral and cellular immune responses without the use of adjuvants [149]. Choi et al. found that mice exposed to oral Stx1B conjugated with monkey rotavirus (SA-11) nonstructural protein NSP4_90_ peptide were protected from rotavirus-mediated gastroenteritis attack. This is due to the strong Th1-mediated response, demonstrating that Stx-B-mediated delivery of viral antigens can elicit both humoral and cellular response in mice [150]. Vingert et al. showed that Stx-B can target dendritic cells in vivo and induce robust and sustained specific CD8+ T-cell responses [54,151].

Stx-B itself has also been utilized to develop vaccines against Stx and EHEC. In 2019, Kordbacheh et al. constructed the HcpA-EspA-Tir-Stx2-B (HETS) vector and immunized BALB/c mice with the purified protein [152]. Stx1-B and Stx2-B have also been fused to *Brucella abortus* lumazine synthase, a highly immunogenic and stable protein that serves as a scaffold to present foreign antigens. The chimeric proteins elicited strong humoral immune responses against different Stx variants in horses. NEAST (neutralizing equine anti-Shiga toxin) is produced using this chimeric antigen and has successfully passed preclinical and Phase I studies. Phase II/III clinical trials are ongoing in Argentina and are recruiting children with bloody diarrhea and Stx2 in feces [153].

Gb3 is also overexpressed in many human cancers [154,155,156,157,158,159,160,161,162,163,164]. Thus, Stx-B has been explored for targeting cancer cells for imaging tumor tissues. Viel and Dransart et al. injected fluorophore-labeled Stx-B intravenously into nude mice xenografted with GFP-expressing HT29 cells and studied its distribution. Stx-B accumulated in HT29 tumor xenografts with high Gb3 expression. In addition, fluorescent Stx-B was found to be slowly cleared through renal excretion. Immunofluorescence staining of the tumor section confirmed that Stx-B not only accumulated in Gb3-positive tumor cells but also entered the epithelial cells of Gb3-expressing neovascularization as well as monocytes and macrophages around the transplanted tumor tissues [54]. Stimmer et al. detected Gb3 expression in primary and metastatic breast cancer cytological specimens as well as human breast cancer xenografts (HBCxs) using Stx-B-CY3 conjugate [156].

Another study by Couture et al. (2011) demonstrated that Stx-B-containing microvesicles can be used to label tumor cells expressing Gb3, which generates hyperechoic signals in ultrasound imaging, and for molecular tumor imaging and to guide ultrasound treatment [165]. For example, Stx-B microvesicles can be used to carry out contrast-enhanced ultrasound imaging of bladder cancer cells that express Gb3 [158,166]. Contrast-enhanced ultrasound technology is already used widely by clinical radiologists, with the advantage of avoiding ionizing radiation [167].

As the direct use of Stx for cancer therapy likely produces high toxicity, several strategies have been developed to utilize Stx-B for the delivery of alternative cytotoxic payloads. The first strategy is to use Stx-B to deliver drugs that are less potent than Stx-A, thus reducing side-effects. For instance, Tarrago-Trani et al. used Stx-B to deliver the photosensitizer e6 chloride (Ce6) to Gb3-positive Vero cells. Ce6 is a second-generation photosensitizer with antitumor activity when used in conjunction with irradiation. Compared with free Ce6, Ce6-Stx-B has been proven to be more effective in delivering Ce6 into target cells [168]. In 2007, Alaoui et al. designed a prodrug Stx-B-SH by conjugating camptothecin SN-38, a camptocamptoid compound that inhibits topoisomerase I. When delivered to Gb3-positive HT-29 colorectal cancer cells and HeLa cells, this compound induces the death of the target cells [169]. A year later, the same group linked RO5-4864, a ligand with high affinity to mitochondrial peripheral benzodiazepine receptors (mPBRs), to Stx-B through a cleavable linker arm. The precursor drug is cleaved intracellularly to release the active ingredient, which improved the cytotoxic activity and antitumor specificity of RO5-4864 in cultured cancer cells [170].

In 2015, Batisse et al. combined Stx-B with a potent auristatin derivative (monomethyl auristatin (MMA)) and optimized the drug conjugation, demonstrating that this targeting system can contribute to selective elimination of Gb3-positive cancer cells in vitro [171]. The same year, Kostova et al. synthesized Stx-B-doxorubicin (StxB-Doxo) and Stx-B-monomethyl auristatin F (StxB-MMAF), two cytotoxic conjugates with strong tumor-suppressive activity in the nanomolar range against HT29 cells [172].

The second strategy is to use protein/peptide cargo targeting cytosolic signaling pathways to replace the highly toxic Stx-A. This can be achieved by fusion of a cargo protein directly to a modified and detoxified Stx-A and then using Stx-B for targeting and delivery into Gb3-positive cells. In 2015, Ryou et al. designed the Shiga-like toxin-based carrier (STC), a delivery system containing Stx-B as the receptor-binding domain and the nontoxic Stx2A^190–297^ as a carrier directly fused with the protein cargo (Figure 3B) [173].

Inspired by the fact that the *Pseudomonas aeruginosa* exotoxin A (PE) translocation domain (the mature form of residues 252 to 364) can be genetically fused to a variety of substances (including proteins, enzymes, and peptides) and efficiently transport them into the cytosol [174], Ryou et al. further improved their STC system by fusing Stx-B to the N-terminus of the *Pseudomonas aeruginosa* exotoxin A (ETA-II, TDP) translocation domain; the C-terminus of TDP was fused to the protein cargo to be delivered. The ER retention sequence (KDEL) was fused to the C-terminus of the cargo for retrograde transport. TDP plays a role in the retrograde transport of the cargo from the ER to the cytosol, and, without TDP, the cargo cannot be released into the cytosol. The authors utilized this system and successfully delivered N8A peptide, which is a mouse double minute 2 (MDM2) inhibitor that blocks the degradation of p53 in cells. The author showed that injection of Stx-B-TDP-N8A suppressed tumor growth in vivo in xenograft mouse models without significant side-effects [175]. The same delivery system has been shown to deliver PEA-15, an ERK kinase regulator, into cells. Nonphosphorylated PEA-15 directly binds to ERK2 and blocks its function, and by cytosolic delivery of PEA-15 mutants with different phosphorylation sites, as well as affinity to ERK2 via the Stx2B-ETAII chimera, the high-affinity mutant was found to have a strong inhibitory effect on cell proliferation by blocking intracellular ERK2 functions [176].

In 2019, Schmit and Neopane et al. further improved the delivery system by linking Stx2B-ETAII with the Von Hippel-Lindau (VHL)-monobody fusion protein. This system targets cancer cells, with the aim of degrading the endogenous tyrosine kinase Lck in Jurkat T-cells and enhancing the inhibition of T-cell receptor (TCR) signaling [177]. VHL is the substrate receptor of the Cullin 2 E3 ligase complex. It can recruit the E3 ligase complex and induce the ubiquitination and degradation of the fusion protein. The monobody in this delivery system can be replaced with any substance that binds an intracellular protein of interest (POI). In combination with VHL, an intracellular POI can be targeted for degradation [177].

Systemic immunogenicity from the toxin protein is one possible limitation to the widespread application of the Stx-B-based delivery system. Possible solutions to this inherent problem include the removal of immunogenic epitopes by protein engineering, encapsulation of the protein [178], and reduction of protein accumulation by local application (e.g., intratumoral injection) [177].

## 9. Conclusions

The *E. coli* outbreak in Germany in 2011, which caused HUS in over 800 patients and 54 deaths, was produced by a new strain, O104:H4 [179]. This strain belongs to enteroaggregative *E. coli* instead of the usual EHEC strains but acquired virulence by acquiring an Stx2 gene, illustrating the continual threat of Stx in causing major outbreaks and the need for developing effective countermeasures. Recent progress in uncovering host factors and establishing a molecular understanding of the biosynthesis of Gb3 has provided new targets for developing inhibitors that can protect cells from Stx. The physiological function and tissue distribution of Gb3 in humans remain a key open question. Recent studies have also begun to reveal the potential role of Stx in taming immune responses and established human intestinal organoid models for understanding how Stx actions may benefit the bacteria. The observation that EHEC T3SS also requires Gb3 is intriguing and deserves further exploration. Excitingly, Stx-based therapeutic protein has entered clinical trials, which will pave the way for broader biomedical applications of toxin-based therapeutics and tools.

## Data Availability

Data sharing not applicable. No new data were created or analyzed in this study. Data sharing is not applicable to this article.
